# Liver fibrosis detection and staging: a comparative study of T1ρ MR imaging and 2D real-time shear-wave elastography

**DOI:** 10.1007/s00261-017-1381-3

**Published:** 2017-12-02

**Authors:** Ruo-kun Li, Xin-pin Ren, Fu-hua Yan, Jin-wei Qiang, Hui-min Lin, Tao Wang, Hong-fei Zhao, Wei-bo Chen

**Affiliations:** 1 Department of Radiology, Ruijin Hospital, Shanghai Jiaotong University School of Medcine, No. 197 Ruijin Er Road, Huangpu District, Shanghai, 200025 China; 2 Department of Ultrasound, Ruijin Hospital, Shanghai Jiaotong University School of Medcine, No. 197 Ruijin Er Road, Huangpu District, Shanghai, 200025 China; 3 Department of Radiology, Jinshan Hospital, Fudan University, No. 1508 Longhang Road, Jinshan District, Shanghai, 201508 China; 4 Philips Healthcare, No. 218 Tianmu Road, Shanghai, China

**Keywords:** Liver fibrosis, T1ρ relaxation elastography, Ultrasonography, Magnetic resonance imaging

## Abstract

**Purpose:**

To compare the results of T1ρ MR imaging and 2D real-time shear-wave elastography (SWE) for liver fibrosis detection and staging.

**Methods:**

Twenty-nine rabbit models of CCl_4_-induced liver fibrosis were established and six untreated rabbits served as controls. T1ρ MR imaging and 2D real-time SWE examination were performed at 2, 4, 6, 8, 10, and 12 weeks. T1ρ values and liver stiffness (LS) values were measured. Fibrosis was staged according to the METAVIR scoring system. Correlation test was performed among T1ρ values, LS values, and fibrosis stage. Receiver operating characteristic (ROC) analysis was performed for assessing diagnostic performance of T1ρ and SWE in detection of no fibrosis (*F*0), substantial fibrosis (≥ *F*2), severe fibrosis (≥ *F*3), and cirrhosis (*F*4).

**Results:**

There was moderate positive correlation between fibrosis stage and T1ρ values (*r* = 0.566; 95% CI 0.291–0.754; *P* < 0.0001), and LS value (*r* = 0.726; 95% CI 0.521–0.851; *P* = 0.003). T1ρ values showed moderate positive correlations with LS values [*r* = 0.693; 95% confidence interval (CI) 0.472–0.832; *P* < 0.0001]. Areas Under ROC (AUROCs) were 0.861 (95% CI 0.705–0.953) for SWE and 0.856 (95% CI 0.698–0.950) for T1ρ (*P* = 0.940), 0.906 (95% CI 0.762–0.978) for SWE and 0.849 (95% CI 0.691–0.946) for T1ρ (*P* = 0.414), 0.870 (95% CI 0.716–0.958) for SWE and 0.799 (95% CI 0.632–0.913) for T1ρ (*P* = 0.422), and 0.846 (95% CI 0.687–0.944) for SWE and 0.692 (95% CI 0.517–0.835) for T1ρ (*P* = 0.137), when diagnosing liver fibrosis with ≥ *F*1, ≥ *F*2, ≥ *F*3, and *F*4, respectively. There was moderate positive correlation between inflammatory activity and T1ρ values (*r* = 0.520; 95% CI 0.158–0.807; *P* = 0.013).

**Conclusion:**

T1ρ imaging has potential for liver fibrosis detection and staging with good diagnostic capability similar to that of ultrasonography elastography.

Liver fibrosis is defined as an abnormal increase in collagen deposition and other components of the extracellular matrix in response to chronic liver injury. It is now well accepted that fibrosis, even early cirrhosis, may regress after successful treatment of the underlying disease (e.g., antiviral therapy in viral hepatitis). Therefore, the stage of liver fibrosis is of paramount importance to determine prognosis and surveillance and to prioritize for treatment and potential for reversibility [1–5].

To date, liver biopsy is still considered the reference standard for fibrosis assessment. However, approximately 25% of patients experience pain during the invasive procedure, and 0.3%–0.6% of patients experience severe complications, such as bleeding and even death [6]. It is also prone to sampling errors and intra- or interobserver variability leading to the over- or understaging. Furthermore, because of its invasive nature, liver biopsy may not be the ideal method for monitoring disease progression and prognostic information [2, 6–10]. Therefore, non-invasive methods, especially imaging technique, have been an intense field of research for liver fibrosis assessment.

Ultrasonography (US) elastography is now widely recognized as a reliable method to assess liver fibrosis. Elastography techniques allow assessment of the viscoelastic or mechanical properties of biologic tissues and provide an estimation of tissue stiffness. Liver fibrosis causes an alteration of the tissue structure, which has an effect on the viscoelastic properties of the respective tissue. Accumulating evidence shows that US elastography, including transient elastography (Fibroscan), acoustic radiation force imaging (ARFI), and shear-wave elastography (SWE), is a reliable method for non-invasive assessment of liver fibrosis [11–15]. Recent studies have shown a correlation between the liver shear modulus measured by using 2D SWE and the degree of fibrosis obtained through liver biopsy [15, 16]. In several meta-analyses, the pooled diagnostic sensitivity and specificity for staging of dichotomized liver fibrosis were 0.80–0.92 and 0.81–0.91, respectively [17–20].

Spin–lattice relaxation time in the rotating frame (T1ρ) represents special MR tissue contrast in biomedical applications. T1ρ is sensitive-to-low frequency motional processes comparable to the Larmor frequency of the spin-lock radiofrequency pulse. It can reflect biologic processes associated with alterations in macromolecular composition and proton exchange in tissues. Until now, T1ρ has been used for diagnosis of tumors, neurodegenerative disease, cartilage degeneration, and myocardial injury [21–24]. Nowadays, whole-liver T1ρ imaging is feasible at 1.5 and 3.0 T, and has potential for fibrosis staging, liver function test, and hepatocyte regeneration evaluation [25–29]. Allkemper et al. found that area under the receiver operating characteristic curve (AUROC) of T1ρ imaging was 0.95–0.98 for the assessment of liver cirrhosis [25]. Wang et al. reported that the liver collagen content is correlated with the degree of elevation of the T1ρ measurements, and T1ρ imaging is able to detect early liver fibrosis in a rat model, indicating that liver T1ρ quantification may play an important role for liver fibrosis diagnosis and staging [30]. In contrast, conflicting results were reported from Takayama et al. on human liver. They found that liver T1ρ relaxation was not significantly correlated with liver fibrosis or with necroinflammation in chronic liver disease [31]. Therefore, further studies are needed to clarify the value of T1ρ imaging for liver fibrosis staging.

In the present study, we aimed to compare the results of T1ρ MR imaging and 2D real-time shear-wave elastography (SWE) for liver fibrosis detection and staging in a rabbit model of CCl4-induced liver fibrosis.

## Materials and methods

### Animal model

This study was approved by the local Institutional Animal Care and Use Committee. Our study group has more experience for liver imaging study using rabbit models [32]. So we choose a rabbit rather than rat liver fibrosis model in the study. A total of 40 male New Zealand white rabbits (2.0–3.0 kg, 4–5 months old) were provided by the CHEDUN Experimental Animal Seed Multiplication Farm of Shanghai. After a week of acclimatization to the standard conditions, these rabbits were randomly distributed into (1) the normal control group (*n* = 6), which were injected with a volume of physiological saline equal to the volume of compounds used in the model group; and (2) the model group (*n* = 34). The rabbit model of CCl_4_-induced liver fibrosis was established with a intraperitoneal injection of 10% (V/V) CCl_4_, at a dose of 0.2 mL/kg of body weight, twice a week. The animals were given the first injection on the same day. Five rabbits in the treatment group died due to poor tolerance of CCl_4_ during the animal establishment period. Finally, 35 rabbits were included in the study. After the first injection, 6, 6, 6, 6, 6, 5, rabbits were selected for MRI at each of six time points of 2, 4, 6, 8, 10, and 12 weeks.

### MRI

MRI was performed using a clinical 3T scanner (Ingenia, Philips Healthcare, Best, the Netherlands) with an eight-channel animal radiofrequency coil (Chenguang Medical Healthcare). The rabbits were anesthetized [3% pentobarbital (w/v)] at 3 mL/kg of body weight by intraperitoneal injection. The abdomen was fixed with a belt to decrease respiratory motion. The standard sequences were performed: (1) axial T2-weighted fast field echo (FFE) (TR/TE = 206/9.2 ms; FOV = 60 × 60 mm; matrix = 100 × 100; slice thickness = 2 mm); (2) axial T1-weighted turbo-spin-echo (TSE) (TR/TE = 400/10 ms; FOV = 60 × 60 mm; matrix = 120 × 93; slice thickness = 2 mm).

For T1ρ imaging, a rotary echo spin-lock pulse was implemented in a 3D turbo field echo (TFE) sequence. Spin-lock frequency was set to 500 Hz, and the spin-lock times (TSL) of 1, 10, 20, 40, and 50 ms were used for T1ρ mapping. Parameters were as follows: TR/TE = 3.8/1.8 ms, flip angle, 40°; slice thickness, 2 mm; voxel size, 1.2 × 1.2 × 2 mm^3^; FOV = 120 × 120 × 10 mm^2^, matrix = 100 × 79, SENSE acceleration factor = 2, TFE factor = 64, NSA = 4, and spectral presaturation inversion recovery fat suppression (SPIR). Five axial slices were selected to cover the central areas of the liver.

T1ρ mapping was conducted using Philips Research Integrated Development Environment (PRIDE) software written in IDL 6.3 (ITT, CO, USA). T1ρ maps were computed on a pixel-by-pixel basis by using a monoexponential decay model, as described by the following equation:

$$M \, \left( {\text{TSL}} \right) = M_{0} \cdot { \exp }\left( { - {\text{TSL}}/{\text{T1}}\rho } \right),$$

where *M* is the signal intensity in the T1rho relaxation preparation images with a certain spin-lock time and TSL is the time of spin-lock pulse.

**Fig. 1:**
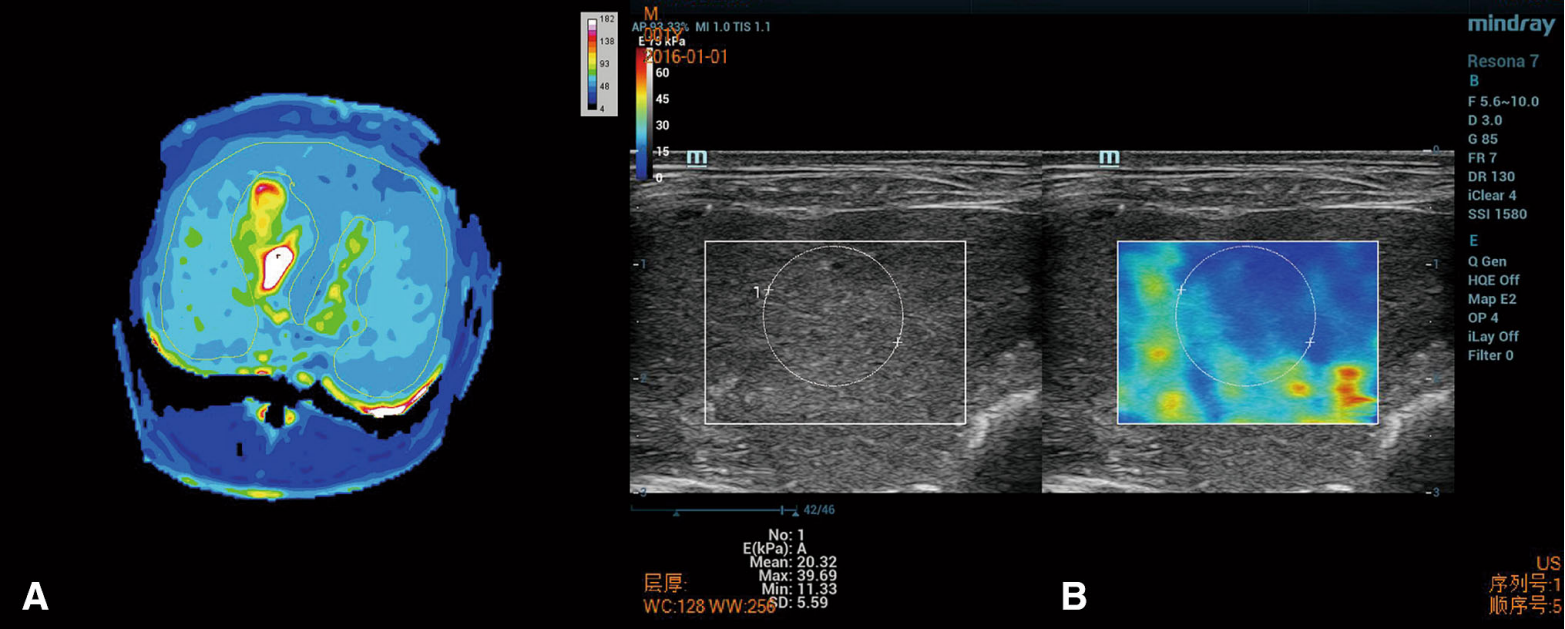
**A** For T1ρ value measurement, ROI was manually drawn conforming to the liver margins but excluding major blood vessels. **B** For LS value measurement, a round ROI was placed in the box on the gray-scale US image in the right liver lobe

Image quantification analyses were conducted using ImageJ software (NIH, Bethesda, MD). T1ρ measurements were performed on the largest section of the liver by a radiologist (A with 5 years of abdominal imaging experience). Regions of interest (ROIs) conforming to the liver margins but excluding major blood vessels were manually drawn on T1ρ maps to measure T1ρ value (Fig. 1A).

### Real-time SWE

2D real-time SWE examination was performed using the Aixplorer US system (Resona 7, mindray, China) with a linear array probe (L15-4) and a frequency of 4–15 MHz by a radiologist (B with 14 years of abdominal imaging experience). The animal was fixed in the supine position. The skin was prepared from the fourth right rib to the abdomen so as to fully expose the examined area. The probe was gently moved without exerting any pressure. Elasticity estimates were color-coded creating a 2D quantitative SWE image of tissue stiffness, which was displayed in box form over a conventional B-mode image. The size and position of the SWE image was user adjustable, enabling a tradeoff in frame rate and extent of view. A round ROI was placed in the box on the gray-scale US image. Liver stiffness (LS) measurements were acquired at least 1 cm below the liver capsule in the right liver lobe. One SWE acquisition may last 5 s approximately. The mean value of six consecutive measurements was used for statistical analyses (Fig. 1B). The breathing has an adverse impact on the LS measurement. So, to achieve stable and reliable data, the image quality is controlled: homogeneous color filling of the color box > 90% and PR of the RLB map > 90%.

### Pathological analysis

After the imaging examination, the animal was immediately euthanized with intravenous injection of 10 mL potassium chloride. The liver tissue samples were immobilized with 4% formalin, and stained with haematoxylin–eosin (HE) and Masson’s trichrome stains.

Liver fibrosis was categorized semi-quantitatively following the METAVIR scoring system as follows: *S*0, no fibrosis; *S*1, enlarged fiber proliferation on portal tracts, and localized perisinusoidal and intralobular fibrosis; *S*2, peripheral fibrosis in the portal area with the formation of fiber septa and intact architecture of the liver lobule; *S*3, fibrous septum accompanied by intralobular structural disorders but without cirrhosis; *S*4, definite cirrhosis. The degree of inflammatory activity was graded on a scale of 0–3 (0 = absent, 1 = mild activity, 2 = moderate activity, 3 = severe activity).

### Statistical analysis

All statistical analyses were performed with commercial software (MedCalc version 12.4.0. Acacialaan, Belgium). Correlation between LS values, T1ρ values, and liver fibrosis stage as well as inflammatory activity was assessed using Spearman’s non-parametric rank correlation coefficient. Pearson correlation test was used to evaluate the correlation of T1ρ values and LS values. Receiver operating characteristic (ROC) analysis was performed for assessing diagnostic performance of T1ρ and SWE in detection of no fibrosis (*F*0), substantial fibrosis (≥ *F*2), severe fibrosis (≥ *F*3), and cirrhosis (*F*4). *P* < 0.05 was considered to indicate a statistically significant result. Optimal cut-off values were decided by maximizing the Youden Index. AUROCs were compared using the DeLong test.

## Results

### Correlation between T1ρ, LS values and fibrosis stage

Histologic fibrosis stages of 35 rabbits were as follows: *F*0, 6; *F*1, 6; *F*2, 7; *F*3, 6; and *F*4, 10. The mean T1ρ value and LS value of *F*0, *F*1, *F*2, *F*3, and *F*4 stage were summarized in Table 1.

**Table 1:** The mean T1ρ value and LS value of different liver fibrosis stages

	*F*0	*F*1	*F*2	*F*3	*F*4
T1ρ (ms)	61.04 ± 9.06	70.8 ± 15.75	83.2 ± 21.22	104.59 ± 25.88	101.07 ± 26.84
LS (kPa)	10.05 ± 1.79	11.12 ± 2.86	16.06 ± 4.66	18.55 ± 5.62	21.52 ± 6.38

**Fig. 2:**
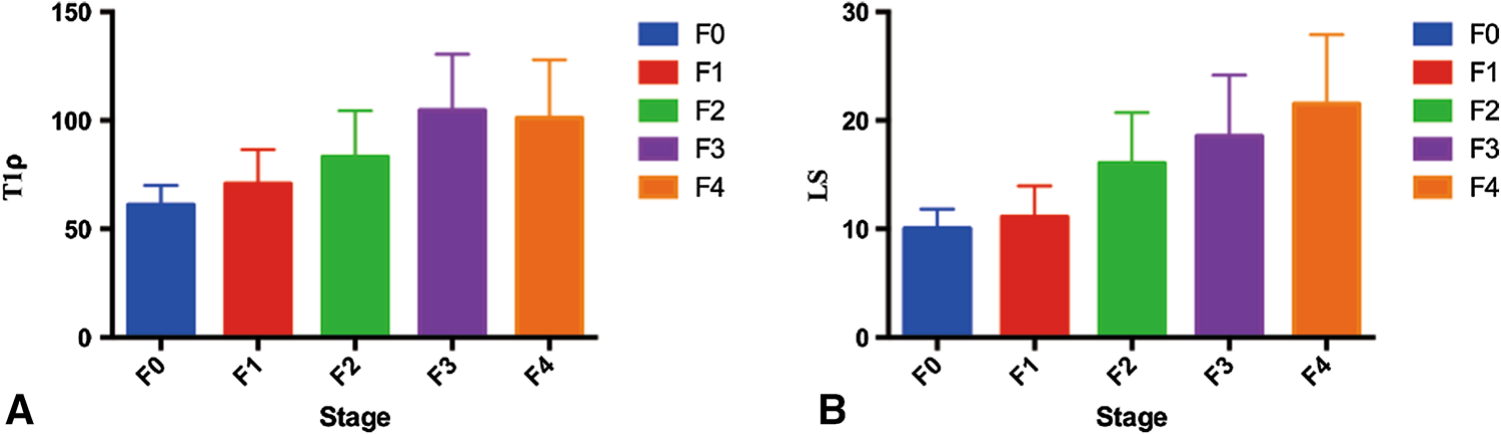
T1ρ (**A**) and LS values (**B**) increased with the severity of liver fibrosis

**Fig. 3:**
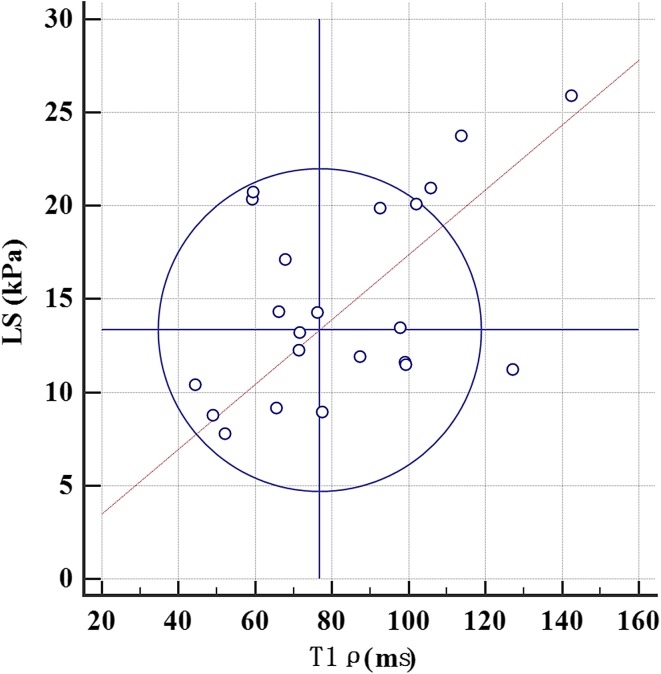
Youden plot graph shows a significant positive correlation between T1ρ and LS values (*r* = 0.693; 95% CI 0.472–0.832; *P* < 0.0001)

**Fig. 4:**
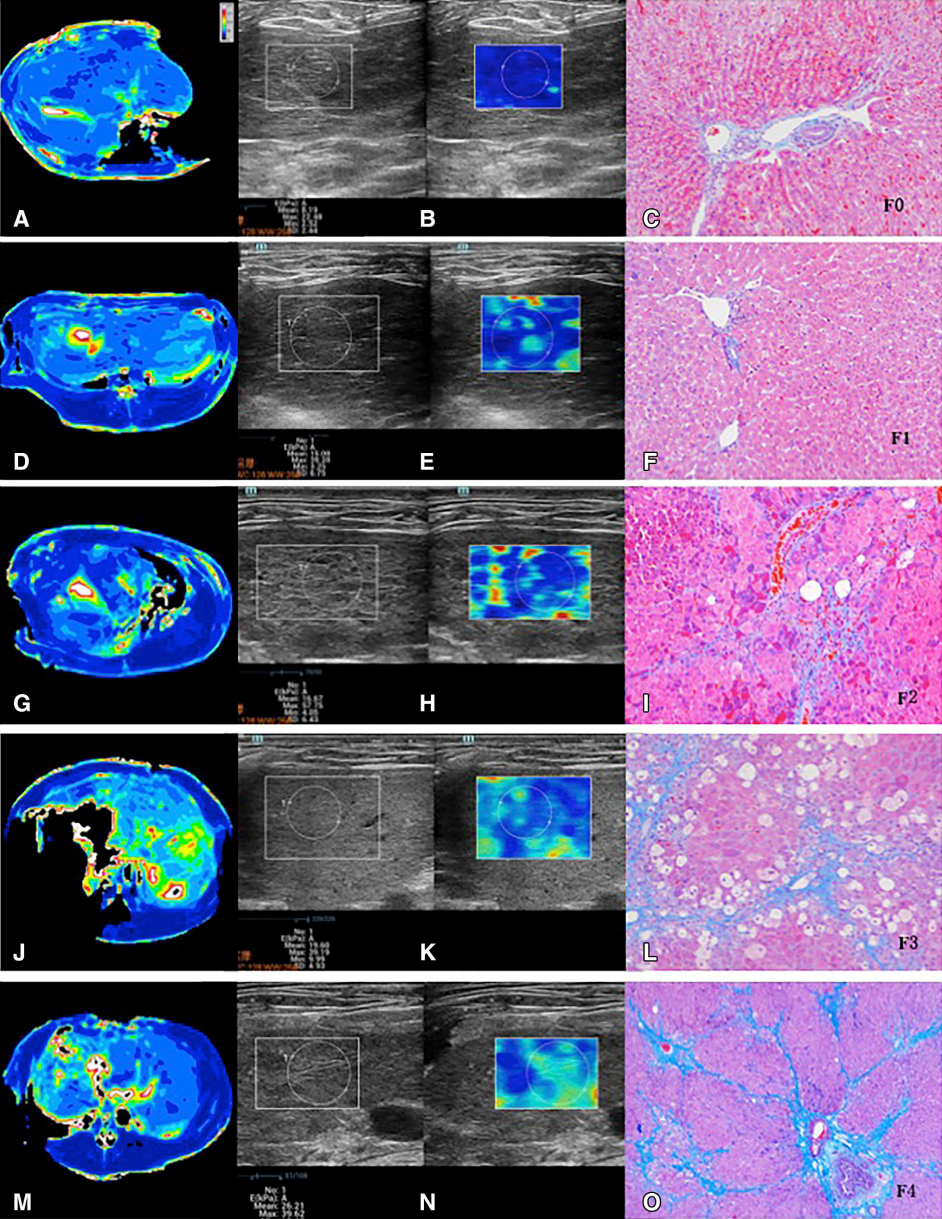
*F*0 (**A**–**C**). **A** T1ρ map, with T1ρ value measurement of 48.98 ms; **B** SWE, with LS value measurement of 8.19 kPa; and Masson trichrome-staining slices (**C** ×100), no fibrosis. F1 (**D**–**F**). **D** T1ρ map, with T1ρ value measurement of 66.04 ms; **E** SWE, with LS value measurement of 15.08 kPa; and Masson trichrome-staining slices (**F** ×100), stage F1 portal and periportal fibrosis. *F*2 (**G**–**I**). **G** T1ρ map, with T1ρ value measurement of 67.85 ms; **H** SWE, with LS value measurement of 16.67 kPa; and Masson trichrome-staining slices (**I** ×100), stage *F*2 fibrosis with few septa. *F*3 (**J**–**L**). **J** T1ρ map, with T1ρ value measurement of 92.43 ms; **K** SWE, with LS value measurement of 19.60 kPa; and Masson trichrome-staining slices (**L** ×100), stage *F*3 fibrosis with bridging and *F*4 (**M**–**O**). **M** T1ρ map, with T1ρ value measurement of 105.76 ms; **N** SWE, with LS value measurement of 26.21 kPa; and Masson trichrome-staining slices (**O** ×100), stage *F*4 fibrosis with overt cirrhosis and nodule formation

The T1ρ and LS values increased along with fibrosis stage (Fig. 2). There was moderate positive correlation between fibrosis stage and T1ρ values (*r* = 0.566; 95% CI 0.291–0.754; *P* < 0.0001), and LS value (*r* = 0.726; 95% CI 0.521–0.851; *P* = 0.003). Moderate positive correlation was also identified between LS value and T1ρ value (*r* = 0.693; 95% CI 0.472–0.832; *P* < 0.0001) (Figs. 3, 4).

### Diagnostic performance of T1ρ and SWE for liver fibrosis staging

**Fig. 5:**
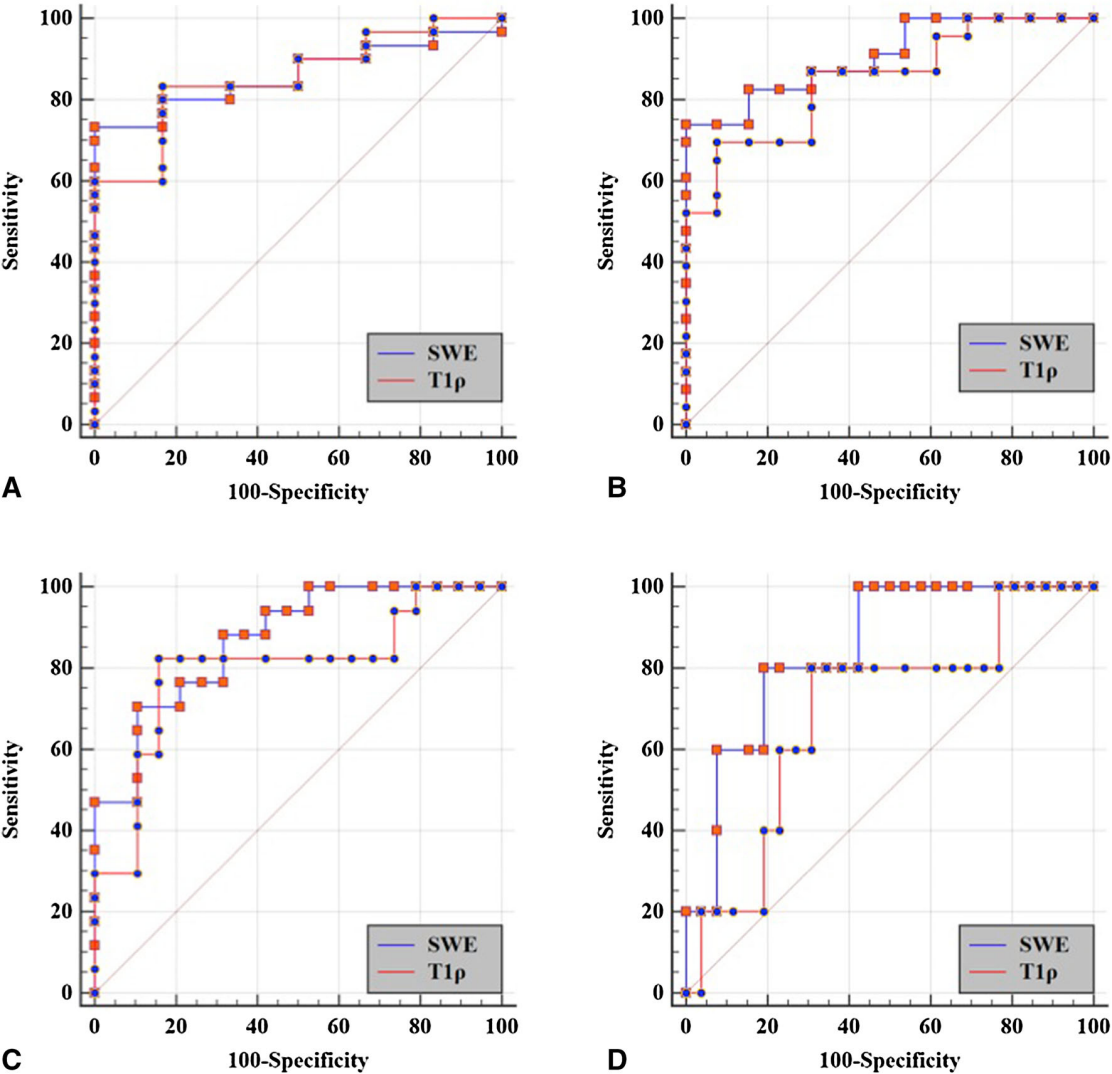
ROC curves of T1ρ and SWE for liver fibrosis staging. **A** F0 versus *F*1–*F*4; **B** *F*0–*F*1 versus *F*2–*F*4; **C** *F*0–*F*2 versus *F*3–*F*4; **D** *F*0–*F*3 versus *F*4

Diagnostic sensitivity and specificity were 69.57%–83.33% and 69.23%–92.31%, respectively, for T1ρ to stage *F*1–*F*4; and 70.59%–80% and 80.77%–100%, respectively, for SWE to stage *F*1–*F*4 liver fibrosis (Table 2). For AUROC comparison, no significant difference was identified for diagnosis of liver fibrosis with different stages (Table 3, Fig. 5).

**Table 2:** Diagnostic performance of T1ρ and SWE for staging liver fibrosis

	≥ *F*1	≥ *F*2	≥ *F*3	*F*4
T1ρ value				
Cut-off value (ms)	62.31	79.45	79.44	92.43
Sensitivity (%)	83.33	69.57	82.35	80
Specificity (%)	83.33	92.31	84.21	69.23
Youden index	0.667	0.619	0.666	0.492
LS				
Cut-off value (kPa)	12.35	14.35	17.12	19.89
Sensitivity (%)	73.33	73.91	70.59	80
Specificity (%)	100	100	89.47	80.77
Youden index	0.733	0.739	0.601	0.608

**Table 3:** Comparison of AUROC of T1ρ and SWE for staging liver fibrosis

	≥ *F*1	≥ *F*2	≥ *F*3	*F*4
T1ρ	0.856 (95% CI 0.698–0.950)	0.849 (95% CI 0.691–0.946)	0.799 (95% CI 0.632–0.913)	0.692 (95% CI 0.517–0.835)
SWE	0.861 (95% CI 0.705–0.953)	0.906 (95% CI 0.762–0.978)	0.870 (95% CI 0.716–0.958)	0.846 (95% CI 0.687–0.944)
*Z* value	0.076	0.818	0.804	1.488
*P* value	0.940	0.414	0.422	0.137

### T1ρ value and inflammatory activity

There was moderate positive correlation between inflammatory activity and T1ρ values (*r* = 0.520; 95% CI 0.158–0.807; *P* = 0.013).

## Discussion

The establishment of new non-invasive approaches is essential in accurately determining the liver fibrosis, and in facilitating the correct diagnosis and monitoring of fibrosis. In the present study, we observed a significant correlation between the T1ρ value, LS value, and the degree of fibrosis in a rabbit model of CCl4-induced liver fibrosis. Additionally, T1ρ imaging demonstrated good diagnostic performance for liver fibrosis staging similar to SWE. To our knowledge, this is the first study that provides a head-to-head comparison of T1ρ imaging and elastography technique.

T1ρ is confirmed to be sensitive to lattice processes occurring at much lower frequency close to the Rabi frequency of the spin-lock radio frequency pulse. Since slow motion in lattice is associated with macromolecular, such as proteins, T1ρ is anticipated to be useful for assessment of the properties of macromolecular environment in tissue. In the articular cartilage study, the loss of proteoglycan results in an increase in the T1ρ relaxation time [33]. Histopathologically, liver fibrosis involves the accumulation of collagen, proteoglycans, and other macromolecules in the extracellular matrix, which may potentially affect T1ρ relaxation. We found that T1ρ could diagnose ≥ *F*1 fibrosis with higher sensitivity (83.33%) than SWE (73.33%), indicating that it may be more sensitive for early detection of liver fibrosis. Our results were in line with that of Wang et al. [30]. They describe the use of T1ρ imaging in the assessment of liver fibrosis in a bile duct ligation model in rats. However, in spite of no significant difference, AUROC of T1ρ (0.692) was slightly lower than that of SWE (0.864), indicating that SWE may be more valuable for cirrhosis detection. This discrepancy may be attributed to imaging mechanism difference for liver fibrosis detection. T1ρ relaxation mainly attributed to macromolecular materials in tissue. Indeed, CCl4-induced diffuse liver fibrosis revealed cellular alterations and histologic patterns, including tissue necrosis, protein transudates, and inflammatory cell infiltration, rather than collagen deposition. We speculate that these pathological changes may be better reflected by T1ρ. In contrast, SWE assesses stiffness indirectly by measuring the speed of shear waves propagating in the tissue of interest. Shear-wave speed is related to tissue stiffness: shear waves travel faster in stiff tissues and slower in soft tissues. We speculate that SWE might be more sensitive for advanced fibrosis or cirrhosis. In addition, small vessel branches could not be accurately excluded due to decreased size in the cirrhotic liver. This may lead to large measurement error and decrease diagnostic performance.

Our study also confirmed a significant positive correlation between T1ρ value and LS value based on 2D SWE. 2D SWE is a novel technique combining real-time visualization of multiple shear waves with traditional ultrasound imaging. It has proven to be efficient for the evaluation of liver fibrosis in clinical and animal studies. This altered response may result from the presence of regenerative nodules and the fibrous bands or septa between them, which can cause distortion, compression, and even obliteration of the hepatic vasculature, or from the increased resistance of the portal venous blood flow and the formation of intrahepatic portosystemic functional shunts. Previous studies demonstrated that liver shear modulus was measured using 2D SWE and the degree of fibrosis was obtained through liver biopsy with good reproducibility measurements [15, 34–38]. A meta-analysis result by Hermann et al. [39] showed that 2D SWE exhibits good to excellent performance for non-invasive staging of liver fibrosis especially in patients with hepatitis B. Overall AUROC of 2D SWE was reported as 0.855–0.955 for liver fibrosis staging, with the pooled sensitivity and specificity of 2D SWE for the diagnosis of liver fibrosis as follows: ≥ *F*1 0.76, 0.92; ≥ *F*2 0.84, 0.83; ≥ *F*3 0.89, 0.86; *F*4 0.89, 0.88, respectively, close to our result (AUROC 0.846–0.906) [39, 40]. In the present study, no significant difference was identified between AUROC of T1ρ (0.692–0.856) and that of LS value (0.846–0.906), indicating that T1ρ had similar diagnostic performance to 2D SWE for liver fibrosis diagnosis and staging. We believe that T1ρ may be feasible for longitudinal research of fibrosis progression from the early stages of liver fibrosis to liver cirrhosis. Further studies are required to move this technology forward and translate it to a clinically applicable imaging biomarker.

So far, transient elastography is the most commonly used elastography method in clinical practice. The main disadvantage of transient elastography is that it is a one-dimensional instantaneous elastography system and does not produce anatomic images, so the precise location of the measurements is not known. Transient elastography is also limited due to ascites, obesity, and operator experience. More recently, 2D-elastography techniques incorporated into conventional US machines (sonoelastography), based on the measurement of the velocity of shear waves generated by mechanically exciting liver tissue by ultrasound pushes, have been introduced. The first described was ARFI followed by SWE. With respect to transient elastography, 2D SWE has the advantage of imaging liver stiffness in real time while guided by a B-mode image, overcomes the attenuation of the adipose tissue on sound velocity, and the region of measurement can be guided with both anatomical and tissue stiffness information [13, 41]. 2D SWE had higher successful rate than transient elastography of liver (98.9% vs. 89.6%) [34]. SWE also had a higher applicability than transient elastography with less impact of inflammation and steatosis, especially in patients with non-advanced fibrosis [36]. Additionally, 2D SWE provides more accurate correlation of liver elasticity with liver fibrosis stage compared with transient elastography, especially in identification of stage *F*2 or greater [15, 34]. Thus, although there is a lack of transient elastography data in our study, we believe that T1ρ imaging is no less valuable than transient elastography for assessment of liver fibrosis.

In the present study, we did not perform MR elastography (MRE) due to lack of imaging equipment and software in our medical center. Therefore, the comparison between T1ρ and MRE cannot be determined. MRE is an MRI-based method for quantitatively imaging tissue stiffness. Compared with US-based elastography techniques, MRE has advantages of larger volume coverage, higher reliable LS measurement rates [42]. However, it remains too cumbersome and not standardized enough for widespread use in routine practice. MRE has been reported to be a useful method for the diagnosis of liver fibrosis. Imajo et al. [43] have compared the accuracy of MRE to that of transient elastography for grading fibrosis in 142 patients with biopsy results. They observed higher AUROC using MRE vs. TE for predicting *F*2–*F*4 fibrosis (0.91 vs. 0.82; *P* = 0.01) and cirrhosis (0.97 vs. 0.92; *P* = 0.49). A good correlation between US- and MR-based ultrasonography has also been determined in previous studies [44, 45]. Yonon [46] compared MRE and SWE for the staging of hepatic fibrosis in the same individuals. Their results demonstrated that LS values from SWE and MRE showed moderate correlation (*r* = 0.724, *P* < 0.001), and LS values measured at SWE were reported as 2.4 times higher than those measured at MRE. SWE and MRE had similar technical success rate (95.35% for MRE, and 97.67% for SWE, *P* = 0.51), and similar diagnostic performance in the diagnosis of liver fibrosis of stage F2 or greater (AUROC 0.853 for MRE; and AUROC 0.852 for MRE; *P* = 0.98). Therefore, despitethe lack of MRE data, we speculate that T1ρ may be no less valuable than MRE for assessment of liver fibrosis.

Our study had several limitations. First, just as mentioned above, MRE was not performed. Further comparative studies should be performed so as to form part of multi-parametric MRI techniques, including T1ρ and MRE, as imaging biomarkers for liver fibrosis evaluation. Second, in our protocol, T1ρ imaging only covered the central areas rather than the whole liver to reduce acquisition time and respiratory artifacts. The imaging technique should be further optimized in the future. Third, we did not use the same imaging plane of liver for SWE and MRI. for LS measurements, we selected the right lobe to represent the liver stiffness of the whole liver. Actually, previous studies have confirmed better accuracy and reproducibility measurements in right lobe than left lobe. In clinical practice, right lobe measurements are also preferred because liver compression by the transducer, heart, or stomach may contribute higher liver stiffness measurements in the left lobe [12, 47–49]. Finally, this study does not provide sufficient histological evaluation of liver fibrosis, and it was difficult to co-register the T1ρ images with the histologic specimens. Heterogeneity of liver fibrosis may have affected the correlation of results between T1p and SWE. However, CCl4-induced fibrosis tends to progress homogeneously. We also selected whole liver rather than special ROI to ensure T1ρ value measurement, which may more accurately reflect T1ρ relaxation changes.

In conclusion, T1ρ imaging has potential for liver fibrosis detection and staging with good diagnostic capability similar to that of ultrasonography elastography.
